# Soil Respiration in Relation to Photosynthesis of *Quercus mongolica* Trees at Elevated CO_2_

**DOI:** 10.1371/journal.pone.0015134

**Published:** 2010-12-06

**Authors:** Yumei Zhou, Mai-He Li, Xu-Bing Cheng, Cun-Guo Wang, A-Nan Fan, Lian-Xuan Shi, Xiu-Xiu Wang, Shijie Han

**Affiliations:** 1 Department of Forest Ecology, Institute of Applied Ecology, Chinese Academy of Sciences, Shenyang, China; 2 Tree Physiology Group, Swiss Federal Research Institute WSL, Birmensdorf, Switzerland; 3 Graduate School of Chinese Academy of Sciences, Beijing, China; 4 Dalian Forest Institute, Dalian, China; 5 School of Life Science, Northeast Normal University, Changchun, China; 6 Research Station of Changbai Mountain Forest Ecosystem, Chinese Academy of Sciences, Erdao, China; University of Zurich, Switzerland

## Abstract

Knowledge of soil respiration and photosynthesis under elevated CO_2_ is crucial for exactly understanding and predicting the carbon balance in forest ecosystems in a rapid CO_2_-enriched world. *Quercus mongolica* Fischer ex Ledebour seedlings were planted in open-top chambers exposed to elevated CO_2_ (EC = 500 µmol mol^−1^) and ambient CO_2_ (AC = 370 µmol mol^−1^) from 2005 to 2008. Daily, seasonal and inter-annual variations in soil respiration and photosynthetic assimilation were measured during 2007 and 2008 growing seasons. EC significantly stimulated the daytime soil respiration by 24.5% (322.4 at EC vs. 259.0 mg CO_2_ m^−2^ hr^−1^ at AC) in 2007 and 21.0% (281.2 at EC vs. 232.6 mg CO_2_ m^−2^ hr^−1^ at AC) in 2008, and increased the daytime CO_2_ assimilation by 28.8% (624.1 at EC vs. 484.6 mg CO_2_ m^−2^ hr^−1^ at AC) across the two growing seasons. The temporal variation in soil respiration was positively correlated with the aboveground photosynthesis, soil temperature, and soil water content at both EC and AC. EC did not affect the temperature sensitivity of soil respiration. The increased daytime soil respiration at EC resulted mainly from the increased aboveground photosynthesis. The present study indicates that increases in CO_2_ fixation of plants in a CO_2_-rich world will rapidly return to the atmosphere by increased soil respiration.

## Introduction

The forest carbon balance is the net result of CO_2_ fixation by aboveground photosynthesis and CO_2_ release, notably the release from the belowground respiration of plant roots, rhizosphere, and soil organisms [1]. Almost 10% of the atmospheric CO_2_ is released by soils each year [2], and this emission is more than the CO_2_ released from fossil fuel combustion [3]. Soil respiration plays, therefore, a crucial role in the global carbon cycle and may be altered strongly by global environmental change [4]–[6].

Some studies found that elevated CO_2_ had no effects on soil respiration [7], [8], or, even suppressed soil respiration [9], [10]. However, many studies using open-top chambers (OTCs) or Free-Air CO_2_ Enrichment (FACE) experiments suggested that elevated CO_2_ led to increased soil respiration rate [4], [5], [11]–[20]. For example, in a northern deciduous forest ecosystem, elevated CO_2_ (534 µmol mol^−1^) significantly stimulated soil respiration (+8–+26%) compared to the controls over three growing seasons [19]. Soil respiration rate was significantly stimulated (+10–+19%) by elevated CO_2_ (580 µmol mol^−1^) throughout 7 years of CO_2_ enrichment in the Duke Forest FACE [18]. Data from four forest FACE experiments have shown that elevated CO_2_ (544 µmol mol^−1^) increased soil respiration by 16 to 39%, and the stimulation persisted for up to 6 years [5].

The rising concentration of atmospheric CO_2_ commonly stimulated both photosynthesis and growth in forest trees [21]–[27], resulting in increased belowground carbon allocation [28], [29]. Increased root biomass and root length stimulated root respiration [15], [30], [31]. After 6-years of CO_2_ enrichment in a mid-successional lowland forest, root biomass and soil respiration increased by 50% and 30%, respectively [32].

There is growing evidence that soil respiration rate is closely correlated with aboveground photosynthesis [1], [33]–[35]. A strong correlation between soil respiration and photosynthesis was observed in a mixed coniferous-deciduous temperate forest [36]. Søe et al. [6] reported that CO_2_ respired by roots (rhizosphere) derived from the recently assimilated CO_2_ accounted for 70% of the total soil respiration at a FACE facility. A large-scale tree-girdling experiment conducted in boreal forests showed that girdling reduced soil respiration by 54% relative to respiration on ungirdled control plots, indicating that current assimilation to roots is a key driver of soil respiration [1]. However, soil respiration does not respond to aboveground photosynthesis in complete synchrony. *In situ* radiocarbon labeling experiment in a black spruce forest revealed that the maximum ^14^C values in roots and rhizosphere respiration occurred 4 days after labeling [37]. Evidence from temperate and boreal forest ecosystems showed that responses of root respiration to canopy photosynthesis lagged for a few hours to three weeks [33], [38].

On the other hand, many environmental factors affect soil respiration [9]. Soil temperature has been recognized to be the most important environmental factor leading to seasonal and diurnal variations in soil respiration; and soil water content was considered to be the secondary variable affecting the temporal variation in soil respiration [39]. Soil respiration rate typically increases exponentially with increasing temperatures, and this relationship is often described using a Q_10_ (magnitude of increase in gas efflux over a 10°C change) [16], [39]–[42]. The Q_10_ values varied greatly with vegetation types and environmental conditions [43]. King et al. [5] reported that Q_10_ values ranged from 1.2 to 4.8 in four FACE experiments with developing and established forests exposed to elevated CO_2_ for 2–6 years. Unlike temperature, there is no common function used to model the relationship between soil respiration and soil moisture. In Siberian tundra systems, soil water significantly affected soil respiration in wet microsites but not in dry microsites [44]. However, the diel variation of soil respiration measured in dry valleys of Antarctica was explained by soil moisture variation [45].

As mentioned above, the responses of soil respiration to elevated CO_2_ gained from the published data seem to vary with the variations of experimental facilities, duration of CO_2_ exposure, plant species, soil property, etc. Hence, we investigated the soil surface respiration rate and plant photosynthesis of *Quercus mongolica* Fischer ex Ledebour plants exposed to elevated CO_2_ in OTCs for three (2007) and four years (2008). The deciduous *Q. mongolica* widely distributed in northern China, Japan, Korea, Mongolia, and eastern Siberia, is a dominant tree species of natural forests in northeastern China. We hypothesize that elevated CO_2_ in deciduous forest ecosystems stimulates soil respiration only during the peak growing season (hypothesis I). The rationale behind this hypothesis is that the photosynthesis of deciduous trees may be significantly affected by elevated CO_2_ only when leaves are expanded but not yellowed. This means that a stimulation of soil respiration may not be detectable during either the early or the late growing season. Hence, a stimulation of soil respiration at elevated CO_2_ in deciduous forests would be attributed mainly to the enhanced aboveground photosynthesis (hypothesis II). In addition, we also hypothesize that the seasonal variation of soil respiration is correlated with soil temperature, but not with soil moisture in the study area (hypothesis III), where the yearly precipitation reaches ∼700 mm.

## Results

### Seasonal variation in soil respiration and photosynthesis

**Figure 1:**
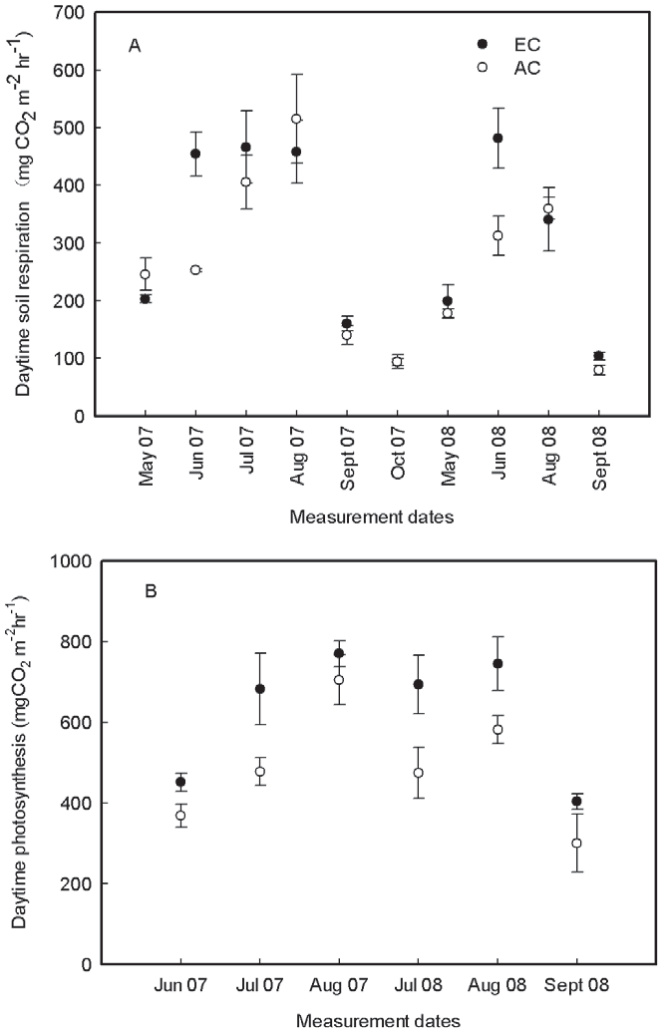
Seasonal and inter-annual variations of accumulated daytime soil respiration (A) and photosynthetic assimilation (B) under elevated CO_2_ (EC) and ambient CO_2_ (AC) during growing seasons in 2007 and 2008. Values are means ±1 SE (*n* = 3).

**Figure 2:**
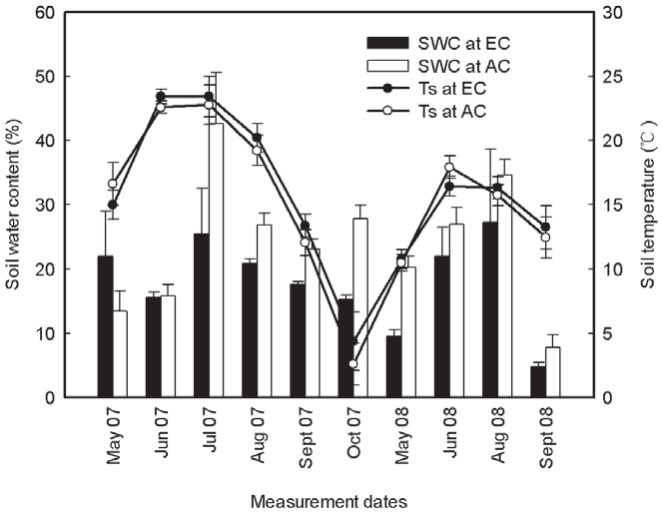
Monthly averaged soil water contents (SWC, *n* = 7; mean ±1 SE) and soil temperature at 5 cm depth (Ts, *n* = 21; mean ±1 SE) at elevated (EC) and ambient CO_2_ (AC) during the experimental period.

Soil respiration showed distinct temporal variation over time (*P*<0.001 for both year and month time scale, Fig. 1A), with the highest values occurring in the peak growing season from June to August (Fig. 1A), when the soil was warm and the soil water content (SWC) was relatively high (Fig. 2). The lower values of accumulated daytime soil respiration occurred in May and October (Fig. 1A) when both soil temperature and soil moisture were low (Fig. 2). The accumulated daytime soil respiration ranged from 93.3 (EC) and 94.4 (AC) mg CO_2_ m^−2^ hr^−1^ in October to 514.6 (EC) and 457.8 (AC) mg CO_2_ m^−2^ hr^−1^ in August 2007. In 2008, the minimum and maximum accumulated daytime soil respiration were 103.9 mg CO_2_ m^−2^ hr^−1^ in September and 481.3 mg CO_2_ m^−2^ hr^−1^ in June at EC, 80.2 mg CO_2_ m^−2^ hr^−1^ in September and 359.9 mg CO_2_ m^−2^ hr^−1^ in August at AC (Fig. 1). No significant differences in soil respiration between EC and AC were found during the early (May) and late growing season (October) (Fig. 1A; *P*>0.05).

Within each CO_2_ treatment, no significant inter-annual and seasonal variations in daytime photosynthesis of trees were found (*P*>0.05; Fig. 1B). The average accumulated CO_2_ assimilation was 624.1 mg CO_2_ m^−2^ hr^−1^ at EC and 484.6 mg CO_2_ m^−2^ hr^−1^ at AC across the two growing seasons.

### Effects of elevated CO_2_ on soil respiration and photosynthesis

Elevated CO_2_ increased the overall accumulated daytime soil respiration significantly (Table 1), especially during the peak of the growing season (June to August) when photosynthetic rate was high and environmental factors, including temperature and moisture, were optimal (Fig. 1A, Fig. 2). The overall percentage increase caused by EC reached 24.5% (322.4 at EC vs. 259.0 mg CO_2_ m^−2^ hr^−1^ at AC) for 2007 and 21.0% (281.2 at EC vs. 232.6 mg CO_2_ m^−2^ hr^−1^ at AC) for 2008, with marked increase of 79.7% (June 2007), 54.1% (June 2008), and 29.6% (September 2008). The average accumulated daytime soil respiration were 306.0 mg CO_2_ m^−2^ hr^−1^ at EC and 248.4 mg CO_2_ m^−2^ hr^−1^ at AC over the two growing seasons (Table 1). However, during the early and late growing season the soil respiration at EC did not significantly differ from that at AC (*P*>0.05, Fig. 1A).

**Table 1:** Effects of CO_2_ treatment on soil respiration rate, photosynthesis, soil temperature at 5 cm depth, soil water content and plant growth (plant height and basal diameter), tested by paired-samples T test.

	Elevated CO_2_	Ambient CO_2_
Soil respiration (mg CO_2_ m^−2^ hr^−1^)	306.0±52.2a	248.4±41.6b
Photosynthesis (mg CO_2_ m^−2^ hr^−1^)	624.1±52.9a	484.6±56.1b
Soil temperature (°C)	16.9±0.6a	16.6±0.6a
Soil water content (% in quality)	18.6±1.9b	23.9±1.7a
Plant height (cm)	106.4±1.2a	86.8±1.1b
Basal diameter (cm)	2.2±0.1a	1.9±0.1b

Different letters between elevated and ambient CO_2_ in each row indicated significant difference at *P*<0.05 level.

Means (± SE) across two growing seasons were given.

Elevated CO_2_ consistently increased daytime CO_2_ assimilation during the measurement period (Fig. 1B, Table 1). On average, elevated CO_2_ increased photosynthesis by 28.8% across the two growing seasons. The highest stimulation percentage of >40% occurred in July for both years. The average values of accumulated CO_2_ assimilation were 624.1 mg CO_2_ m^−2^ hr^−1^ at EC and 484.6 mg CO_2_ m^−2^ hr^−1^ at AC over the two growing seasons (*P*<0.01; Table 1).

### Responses of soil respiration to soil temperature and soil moisture

No difference in soil temperature at a depth of 5 cm (T_s_) between CO_2_ treatments was detected (Tables 1 and 2; Fig. 2). T_s_ exhibited pronounced seasonal fluctuation (*P*<0.001) and inter-annual variation (*P*<0.001) (Fig. 2). Patterns of changes in T_s_ were similar between the two CO_2_ treatments (Fig. 2).

**Table 2:** Temperature response functions of soil respiration (µmol m^−2^s^−1^) to soil temperature (°C); soil temperature and soil water content (% in quality) during growing seasons in 2007 and 2008.

	Fitted equation	R^2^	Q_10_	T_s_ (Min)	T_s_ (Max)	T_s_ (Mean)	SWC (Min)	SWC (Max)	SWC (Mean)
2007									
Elevated CO_2_	SR = 0.7096 exp (0.0822 T_s_)	0.7776	1.62±0.03a	4.4±2.3	23.4±0.6	16.6±2.9a	15.2±0.7	25.4±7.1	19.4±1.7a
Ambient CO_2_	SR = 0.8243 exp (0.0641 T_s_)	0.7225	1.56±0.08a	2.6±1.6	22. 8±1.6	16.0±3.1a	13.4±3.2	42.6±7.9	24.9±4.3b
2008									
Elevated CO_2_	SR = 0.3707 exp (0.1438T_s_)	0.7390	1.54±0.16a	10.8±0.7	16. 5±0.8	14.2±1.4a	4.8±0.7	26.1±4.8	17.9±4.5a
Ambient CO_2_	SR = 0.4506 exp (0.1250 T_s_)	0.7736	1.57±0.05a	10.5±0.6	17.9±0.9	14.2±1.7a	7.7±2.1	34.6±2.4	22.2±9.9b

*SR* soil respiration rate, *T_s_* soil temperature, *SWC* soil water content.

Different letters between elevated and ambient CO_2_ within the same year and the same category indicated significant difference at *P*<0.05 level (*n* = 3).

**Figure 3:**
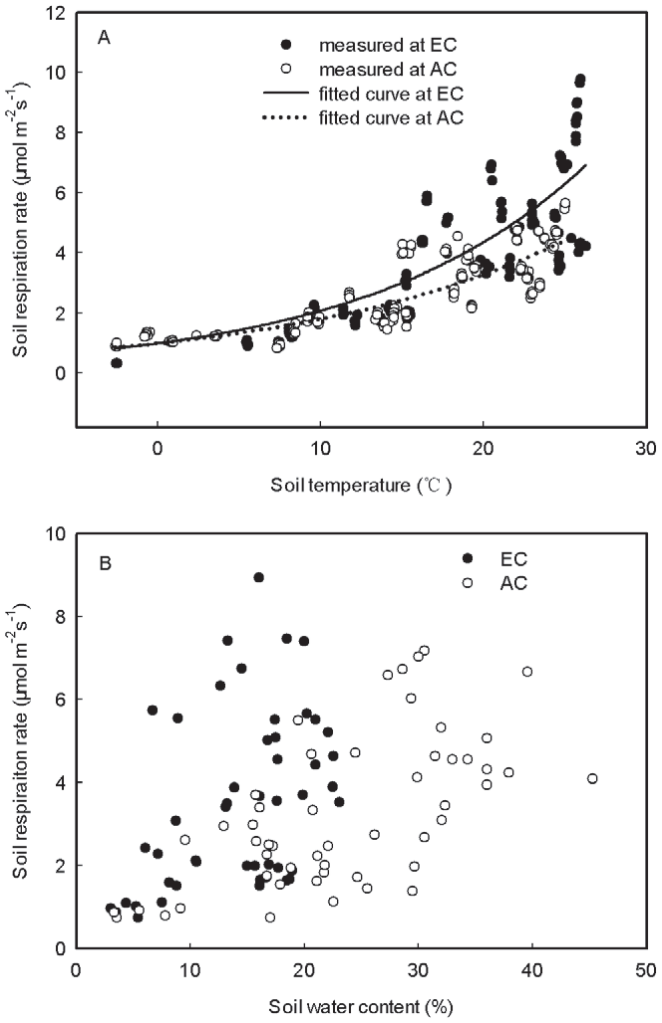
Soil respiration in relation to soil temperature at 5 cm depth (A) and soil water content (B) at elevated (EC) and ambient CO_2_ (AC) during growing seasons. Trend lines were generated for each CO_2_ treatment using exponential regression.

Soil respiration increased exponentially with T_s_ for both CO_2_ treatments (Fig. 3A). T_s_ explained >72% of the variations in soil respiration (Table 2). No significant difference in Q_10_ values between the two CO_2_ treatments was detected for both 2007 and 2008 (*P*>0.05; Table 2). Across the two growing seasons, the Q_10_ values were about 1.6 for both CO_2_ treatments (Table 2).

Soil respiration increased with increasing SWC during the growing season for both CO_2_ treatments, showing a positive correlation between soil respiration and SWC (Fig. 3B, Table 3). SWC at EC exhibited similar seasonal trend to AC and peaked in July and August (Fig. 2). In 2007, SWC ranged from 15.2% in October to 25.4% in July (19.4% on average) at EC and from 13.4% in May to 42.6% in July (24.9% on average) at AC (Table 2). In 2008, the mean SWC was 17.0% at EC with the lowest value of 4.8% in September and the highest value of 26.1% in July, whereas the mean SWC was 22.2% at AC with the lowest value of 7.7% in September and the highest value of 34.6% in August (Table 2). Elevated CO_2_ significantly decreased SWC (Table 1) by 11.7% in 2007 and 20.6% in 2008.

**Table 3:** The Pearson's correlation coefficient among soil respiration, photosynthesis, plant height, basal diameter, soil temperature at 5 cm depth and soil water content at elevated and ambient CO_2_ during growing seasons in 2007 and 2008.

	SR	*P*_N_	H	D	Ts	SWC
SR	1.000					
*P*_N_	0.226*	1.000				
H	−0.067	0.153	1.000			
D	−0.229	0.246	0.956**	1.000		
Ts	0.678**	0.024	−0.245	−0.341	1.000	
SWC	0.436**	0.169	−0.075	−0.107	0.239*	1.000

*SR* soil respiration, *P*_N_ photosynthesis, *H* plant height, *D* basal diameter, *Ts* soil temperature at 5 cm depth, *SWC* soil water content

*indicates correlation is significant at 0.05 level and

**at 0.01 level (2-tailed).

### Growth responses

**Figure 4:**
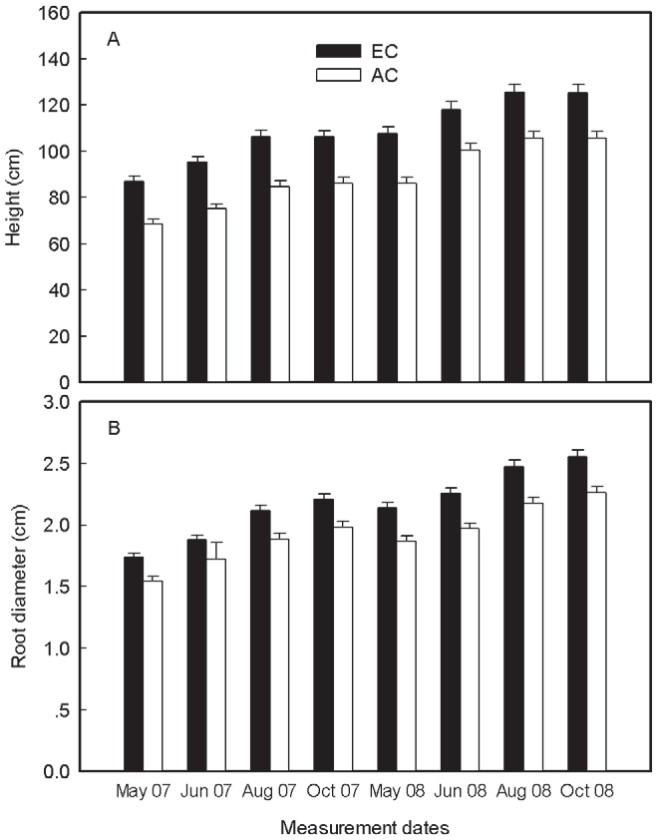
Growth (mean ±1 SE) of plant height (A) and basal diameter (B) under elevated and ambient CO_2_ during growing seasons in 2007 and 2008.

The plant height and basal diameter at EC were significantly greater than those at AC across the two growing seasons (Fig. 4, Table 1). The plants stop growing after October until next May; therefore, the height and basal diameter in October 2007 were similar to those in May 2008 (Fig. 3). The plant height increased by 19.2 cm and 17.6 cm at EC and 17.6 cm and 19.6 cm at AC for 2007 and 2008, respectively. Similarly, the mean basal diameter increased by 0.4 cm at EC and 0.3 cm at AC for each year.

### Relationships between soil respiration and biotic and abiotic factors

Daytime soil respiration was positively correlated with T_s_ (*P*<0.01), SWC (*P*<0.01), and photosynthetic assimilation (*P*<0.05) for both AC and EC treatment (Table 3). In addition, positive correlation between T_s_ and SWC was also found during the two growing seasons (Table 3).

## Discussion

The seasonal pattern of soil respiration in our study showed that soil respiration was higher during the middle growing season just when the soil temperature, soil moisture and photosynthesis were also higher. These results are consistent with the findings of Niinistö et al. [16] and Pregitzer et al. [46], who found that the greatest soil respiration in forest ecosystems occurred during the peak growing season, and the lowest soil respiration was measured in spring and autumn when soil temperatures was relatively low and the canopy density was low.

Previous studies found that the increase magnitude of soil respiration caused by elevated CO_2_ varied greatly with the tree species or vegetation cover [5], [12], [19]. The present study with deciduous *Q. mongolica* under elevated CO_2_ showed a monthly increase in soil respiration by 23.0%. Similarly in deciduous forests, elevated CO_2_ has been found to stimulate soil respiration by 39% in stands of paper birch and trembling aspen [15], and 8–26% in aspen forests [19]. In evergreen forests, elevated CO_2_ stimulated the soil respiration by 24% in loblolly pine (*Pinus taeda* L.) plantation [18], 50–57% in ponderosa pine [11], [12], 23–37% in 20-year-old Scots pines (*Pinus sylvestris* L.) [16], and 20% in Douglas-fir (*Pseudotsuga menziesii* (Mirb.) Franco) seedlings [47].

Consistent with our hypothesis I (see Introduction), the present study found that elevated CO_2_ significantly stimulated soil respiration from June to August (Fig. 1A, Table 1) and this stimulation was significantly correlated with enhanced photosynthesis (Fig. 1, Table 3). Increased plant height and basal diameter under elevated CO_2_ implied higher root biomass. Thus, the increase in respiring roots and photosynthate availability at elevated CO_2_ may result in pronounced increase in soil respiration compared to ambient CO_2_. Increased root biomass and production at elevated CO_2_ have already been recognized to be the most common reason resulting in increased soil respiration [12], [16], [19], [48]–[51]. Previous studies indicated that there was a close relationship between aboveground photosynthesis and soil respiration since root respiration consumes recently fixed photosynthates from foliages [1], [13], [46], [52]–[54]. Andrews and Schlesinger [53] attributed the increase in forest soil respiration to increased root and rhizosphere respiration under elevated CO_2_. Pregitzer et al. [19] reported that the recently fixed carbon by photosynthesis accounted for 60–80% of soil respiration during the peak growing season. Although there are still uncertainties regarding the relative contribution of roots or rhizosphere to the total soil respiration in the present study, higher soil respiration associated with higher photosynthesis under elevated CO_2_ may imply a greater carbon input to roots/rhizosphere. Thus, consistent with our hypothesis II (see Introduction), the increase in soil respiration at elevated CO_2_ is mainly attributed to increased aboveground photosynthesis.

Soil respiration varied significantly with year and month (*P*<0.05), which may be a combined result of temporal variation in soil temperature, aboveground photosynthesis, and root growth rate. King et al. [5] found that the inter-annual variation of soil respiration in four FACE systems was determined by changes in soil temperature influencing plant photosynthesis and root growth. Regardless of CO_2_ treatment, soil respiration increased exponentially with soil temperature [29], [55], [56]. Variations in soil temperature alone accounted for over 70% of the variation in soil respiration at both elevated and ambient CO_2_ found in our study (Table 2). This finding is comparable to past work which found that soil temperature explained 70% of the variation in soil respiration in a *Pinus cembra* forest [57], 72% of the variation in a spruce forest [58], and 80% of the variation in a mixed temperate hardwood forest [59].

Elevated CO_2_ did not change the temperature sensitivity of soil respiration since no significant difference in Q_10_ values between elevated and ambient CO_2_ was found (Table 2). Similarly, King et al. [5] also found that the temperature sensitivity of soil respiration appeared to be unaffected by elevated CO_2_. However, soil respiration rates were found to be more sensitive to changes in soil temperature at elevated CO_2_ than at ambient CO_2_ [9]. For example, the Q_10_ values were 2.4 in ambient CO_2_ versus 2.8 in elevated CO_2_ in a ponderosa pine forest [9], 1.5 to 3.2 at ambient CO_2_ and 1.2 to 4.8 at elevated CO_2_ in a *Populus* plantation [5], 1.9 to 3.3 in a mixed temperate forest [60]. The Q_10_ values of ∼1.6 for both CO_2_ treatments found in the present study (Table 2) are similar to the values of 1.4 to 1.8 found in a ponderosa pine plantation [61]. The temperature sensitivity of soil respiration has been recognized to be positive with substrate supply [9]. Elevated CO_2_ increased the carbon allocation to roots, providing additional substrates for root respiration [62], [63], and further leading to an increased temperature sensitivity of root respiration and soil respiration. On the other hand, decreased soil moisture resulted from elevated CO_2_ can result in a decrease in activity and biomass of soil organisms [64], consequently causing reduction in soil microbial respiration and soil respiration. In the present study, no apparent CO_2_ effects on Q_10_ were found (Table 2). This result may be resulted from an interaction between elevated CO_2_ and soil moisture since elevated CO_2_ increased the aboveground photosynthesis and root respiration (Fig. 1), but decreased the soil moisture (Fig. 2).

The contribution of soil water content to soil respiration was found to depend on vegetation cover and soil properties [39], [44], [45]. The mathematical relationship between soil respiration and soil moisture is relatively complex if a correlation between them exists [65], [66]. In six forest plantations located at Rwanda in African, soil respiration is mainly related to soil water content which explained 36–77% of the temporal variation in soil respiration [67]. The quadratic relationship between soil respiration and soil water content in a tallgrass prairie accounted for 26% of the variation in soil respiration [65]. Soil moisture is also considered as an important factor affecting soil respiration in our study because a significantly positive correlation between soil water content and soil respiration has been detected (Table 3). This finding is consistent with the results gained in a semiarid grassland ecosystem [68], but did not support our hypothesis III (see Introduction). Elevated CO_2_ decreased soil water content in the present study (Table 1, Fig. 2), probably due to greater water loss caused by greater leaf area. Since the precipitation in the research area mainly occurs during the peak growing season (from June to August) when temperature is also relatively higher, soil water content is then positively correlated with soil temperature in our study (Table 3).

### Conclusion

An enhanced photosynthetic assimilation leading to increased plant growth at elevated CO_2_ implies greater root respiration consuming the recently fixed carbon. Increased photosynthetic assimilation and aboveground growth may create dense canopy to fix more carbon but also stimulate belowground respiration. Hence, the present study indicates that increase in CO_2_ fixation of plants in a CO_2_-rich world will rapidly return to the atmosphere by increased soil respiration.

## Materials and Methods

### Research area and experiment design

The experiment was conducted at the Research Station of Changbai Mountain Forest Ecosystem (42°24′ N, 128°05′ E, 738 m a.s.l.), Jilin Province, northeastern China. The annual mean air temperature is 3.6°C and annual mean precipitation is 695 mm [69]. The maximum air temperature and over 60% precipitation occurred in June to August. Ten hexagon OTCs (4.0 m in both height and diameter) with clear glass were established nearby the research station. Uniform local forest soil with a total organic carbon of 9.0% was used in OTCs [70]. Three-year-old plants, with similar plant size in height and basal diameter, of *Q. mongolica* from a nearby plantation were planted at a spacing of 0.5×0.5 m in OTCs in spring 2005.

CO_2_ fumigation treatments (AC  =  ambient CO_2_ concentration of 370 µmol mol^−1^CO_2_ for 4 chambers, and EC  =  elevated CO_2_ concentration of 500 µmol mol^−1^CO_2_ for the other 6 chambers) began in 2005. Elevated CO_2_ was supplied in daytime during each growing season from the beginning of May to the end of October. Elevated CO_2_ has been supplied to the chambers by pipes connected to industrial CO_2_ tanks outside the chambers. The concentrated CO_2_ was pumped into the chambers from a height of 1.6 m and was diffused. The CO_2_ concentration (500±100 µmol mol^−1^CO_2_) was recorded every 10 min by CO_2_ sensors (SenseAir, Sweden) installed in the center of each chamber and automatically adjusted to the target concentration by controlling the input amount of concentrated CO_2_. All chambers accepted natural rainfall during the experimental period.

### Soil respiration measurements

The soil respiration presented in this study is defined as an integrating respiration of all components including root respiration, microbial and soil fauna respiration, and chemical process emission, but excluding the respiration of litter. Soil surface respiration was measured from 6:00 am to 6:00 pm at a two-hours' interval from May to October in 2007 and repeated in 2008, using a LI-6400 portable photosynthesis system (LI-6400, LI-COR, Lincoln, NE, USA) in closed circuit with a LI-6400-09 soil respiration chamber (SRC). The measurements were carried out according to the methods described in detail by Tingey et al. [9]. Soil respiration collars, constructed from PVC pipe, were randomly distributed in the center and on the edge of each OTC. We did not find any significant difference in soil respiration measured on the edge of OTCs compared to that measured in the chamber center. Collars remained in the same measurement locations throughout the measurement. The collars were inserted approximately 2 cm into the soil depth, and matched well with the SRC to avoid possible leakage. All litter and herbs were carefully removed during the experiment period. To ensure parallel measurements and to diminish the effects of environmental variations of temperature and humidity on parameters measured, we selected two points (measurement locations) at each chamber, and three chambers per treatment only.

The T_s_ was recorded with soil respiration rate concurrently by a portable temperature probe attached to the analyzer which was inserted into the soil adjacent to the SRC. The 0–5 cm soil layer under each SRC was cored after each measurement. The soil was weighed before and after dried at 100°C for 48h. The SWC was expressed as a percentage of water mass to dry soil mass. The soil respiration rate per treatment for each measurement time was based on the average of the data from the two locations in three OTCs.

The soil respiration was measured only during the daytime from 6 am to 6 pm corresponding to the photosynthesis measurements.

### Photosynthesis and growth measurements

The daily course of photosynthesis was also measured concurrently during the daytime from 6 am to 6 pm at a two-hours' interval on clear days during the growing seasons in 2007 and repeated in 2008. Photosynthetic measurements were conducted using the same system of LI-6400 (LI-COR, Lincoln, NE, USA). All measurements were made *in situ* on fully sunlit leaves in trees at respective growth CO_2_ concentrations (AC, EC). Plant height and basal diameter of all individuals in each OTC were measured during early, peak and late growing season each year.

### Q_10_ of soil respiration

Scatter plots were used to determine the relationship between soil respiration and T_s_ for 2007 and 2008, respectively, using the data gained from a complete growing season (May - October) in each year. The scatter plots were fitted by an exponential relationship between measured data of soil respiration rate (*y*) and T_s_ (*x*): *y*  =  *a*e*^bx^*, where *a* and *b* are coefficients. The Q_10_ values were then calculated using Q_10_  =  e^10*b*^.

### Estimating accumulated daytime soil respiration and CO_2_ assimilation

Accumulated daytime soil respiration was obtained by integrating values measured during the daytime for each treatment. Integrated values were calculated by determining the area under each segment of two consecutive measurement points and then summing the segments for a total daytime soil respiration. For a detailed method description, see Vose et al. [12]. The same method was also applied for calculating the daytime CO_2_ assimilation, using the daily course of net photosynthetic rate.

### Statistical analyses

Statistical analyses were performed with SPSS 13.0 software program (SPSS Inc, 2004). The normality test was carried out using P-P test on datasets prior to statistical analyses to verify a normal distribution. We used paired-samples T test to test the differences in soil respiration, photosynthesis, soil temperature at 5 cm depth, soil water content, and plant growth in height and basal diameter between EC and AC within each measurement date. One-way ANOVA was used to compare the difference in Q_10_ values between EC and AC. The Pearson correlation was used to detect the correlation among soil respiration, photosynthesis, tree growth and environmental factors.

## References

[1] Högberg P, Nordgren A, Buchmann N, Taylor AFS, Ekblad A (2001). Large-scale forest girdling shows that current photosynthesis drives soil respiration.. Nature.

[2] Raich JW, Potter CS (1995). Global patterns of carbon dioxide emissions from soils.. Global Biogeochemical Cycles.

[3] Raich JW, Tufekcioglu A (2000). Vegetation and soil respiration: correlations and controls.. Biogeochemistry.

[4] Zak DR, Pregitzer KS, King JS, Holmes WE (2000). Elevated atmospheric CO_2_, fine roots and the response of soil microorganisms: a review and hypothesis.. New Phytologist.

[5] King JS, Hanson PJ, Bernhardt E, Deangelis P, Norby RJ (2004). Multiyear synthesis of soil respiration responses to elevated atmospheric CO_2_ from four forest FACE experiments.. Global Change Biology.

[6] Søe ARB, Giesemann A, Anderson T-H, Weigel H-J, Buchmann N (2004). Soil respiration under elevated CO_2_ and its partitioning into recently assimilated and older carbon sources.. Plant and Soil.

[7] Thomas SM, Cook FJ, Whitehead D (2000). Seasonal soil-surface carbon fluxes from the root systems of young *Pinus radiate* trees growing at ambient and elevated CO_2_ concentration.. Global Change Biology.

[8] Daly E, Palmroth S, Stoy P, Siqueira M, Oishi AC (2009). The effects of elevated atmospheric CO_2_ and nitrogen amendments on subsurface CO_2_ production and concentration dynamics in a maturing pine forest.. Biogeochemistry.

[9] Tingey DT, Johnson MG, Lee EH, Wise C, Waschmann R (2006). Effects of elevated CO_2_ and O_3_ on soil respiration under ponderosa pine.. Soil Biology and Biochemistry.

[10] Tingey DT, Lee EH, Waschmann R, Johnson MG, Rygiewicz PT (2006). Does soil CO_2_ efflux acclimatize to elevated temperature and CO_2_ during long-term treatment of Douglas-fir seedlings?. New Phytologist.

[11] Johnson D, Geisinger D, Walker R, Newman J, Vose J (1994). Soil *p*CO_2_, soil respiration, and root activity in CO_2_-fumigated and nitrogen-fertilized ponderosa pine.. Plant and Soil.

[12] Vose JM, Elliott KJ, Johnson DW, Walker RF, Johnson MG (1995). Effects of elevated CO_2_ and N fertilization on soil respiration from ponderosa pine (*Pinus ponderosa*) in open-top chambers.. Canadian Journal of Forest Research.

[13] Luo Y, Jacson RB, Field CB, Mooney HA (1996). Elevated CO_2_ increases belowground respiration in California grasslands.. Oecologia.

[14] Vose JM, Elliott KJ, Johnson DW, Tingey DT, Johnson MG (1997). Soil respiration response to three years of elevated CO_2_ and N fertilization in ponderosa pine (*Pinus ponderosa* Doug. ex Laws.).. Plant and Soil.

[15] King JS, Pregitzer KS, Zak DR, Sober J, Isebrands JG (2001). Fine-root biomass and fluxes of soil carbon in young stands of paper birch and trembling aspen as affected by elevated atmospheric CO_2_ and tropospheric O_3_.. Oecologia.

[16] Niinistö SM, Silvola J, Kellomäki S (2004). Soil CO_2_ efflux in a boreal pine forest under atmospheric CO_2_ enrichment and air warming.. Global Change Biology.

[17] Barron-Gafford G, Martens D, Grieve K, Biel K, Kudeyarov V (2005). Growth of Eastern Cottonwoods (*Populus deltoides*) in elevated [CO_2_] stimulates stand-level respiration and rhizodeposition of carbohydrates, accelerates soil nutrient depletion, yet stimulates above- and belowground biomass production.. Global Change Biology.

[18] Bernhardt ES, Barber JJ, Pippen JS, Taneva L, Andrews JA (2006). Long-term effects of free air CO_2_ enrichment (FACE) on soil respiration.. Biogeochemistry.

[19] Pregitzer K, Loya W, Kubiske M, Zak D (2006). Soil respiration in northern forests exposed to elevated atmospheric carbon dioxide and ozone.. Oecologia.

[20] Taneva L, Gonzalez-Meler MA (2008). Decomposition kinetics of soil carbon of different age from a forest exposed to 8 years of elevated atmospheric CO_2_ concentration.. Soil Biology and Biochemistry.

[21] Liu S, Teskey RO (1995). Responses of foliar gas exchange to long-term elevated CO_2_ concentrations in mature loblolly pine trees.. Tree Physiology.

[22] Curtis PS, Vogel CS, Wang XZ, Pregitzer KS, Zak DR (2000). Gas exchange, leaf nitrogen, and growth efficiency of *Populus tremuloides* in a CO_2_-enriched atmosphere.. Ecological Applications.

[23] Sholtis JD, Gunderson CA, Norby RJ, Tissue DT (2004). Persistent stimulation of photosynthesis by elevated CO_2_ in a sweetgum (*Liquidambar styraciflua*) forest stand.. New Phytologist.

[24] Ainsworth EA, Long SP (2005). What have we learned from 15 years of free-air CO_2_ enrichment (FACE)? A meta-analytic review of the responses of photosynthesis, canopy properties and plant production to rising CO_2_.. New Phytologist.

[25] Rasse DP, Peresta G, Drake BG (2005). Seventeen years of elevated CO_2_ exposure in a Chesapeake Bay Wetland: sustained but contrasting responses of plant growth and CO_2_ uptake.. Global Change Biology.

[26] Liberloo M, Tulva I, Raim O, Kull O, Ceulemans R (2007). Photosynthetic stimulation under long-term CO_2_ enrichment and fertilization is sustained across a closed *Populus* canopy profile (EUROFACE).. New Phytologist.

[27] Aranjuelo I, Pardo A, Biel C, Savé R, Azcón-Bieto J (2009). Leaf carbon management in slow-growing plants exposed to elevated CO_2_.. Global Change Biology.

[28] Jach ME, Laureysens I, Ceulemans R (2000). Above- and below-ground production of young Scots pine (*Pinus sylvestris* L.) trees after three years of growth in the field under elevated CO_2_.. Annuals of Botany.

[29] Butnor JR, Johnsen KH, Oren R, Katul GG (2003). Reduction of forest floor respiration by fertilization on both carbon dioxide-enriched and reference 17-year-old loblolly pine stands.. Global Change Biology.

[30] Griffin KL, Bashkin MA, Thomas RB, Strain BR (1997). Interactive effects of soil nitrogen and atmospheric carbon dioxide on root/rhizosphere carbon dioxide efflux from loblolly and ponderosa pine seedlings.. Plant and Soil.

[31] Janssens IA, Crookshanks M, Taylor G, Ceulemans R (1998). Elevated atmospheric CO_2_ increases fine root production, respiration, rhizosphere respiration and soil CO_2_ efflux in Scots pine seedlings.. Global Change Biology.

[32] Ball AS, Milne E, Drake BG (2000). Elevated atmospheric-carbon dioxide concentration increases soil respiration in a mid-successional lowland forest.. Soil Biology and Biochemistry.

[33] Tang J, Baldocchi D, Xu L (2005). Tree photosynthesis modulates soil respiration on a diurnal time scale.. Global Change Biology.

[34] Hartley IP, Armstrong AF, Murthy R, Barron-Gafford G, Ineson P (2006). The dependence of respiration on photosynthetic substrate supply and temperature: integrating leaf, soil and ecosystem measurements.. Global Change Biology.

[35] Bahn M, Schmitt M, Siegwolf R, RichterAndreas, Brüggemann N (2009). Does photosynthesis affect grassland soil-respired CO_2_ and its carbon isotope composition on a diurnal timescale?. New Phytologist.

[36] Sampson DA, Janssens IA, Yuste JC, Ceulemans R (2007). Basal rates of soil respiration are correlated with photosynthesis in a mixed temperate forest.. Global Change Biology.

[37] Carbone MS, Czimczik CI, McDuffee KE, Trumbore SE (2007). Allocation and residence time of photosynthetic products in a boreal forest using a low-level ^14^C pulse-chase labeling technique.. Global Change Biology.

[38] Davidson EA, Holbrook NM, Noormets A (2009). Is temporal variation of soil respiration linked to the phenology of photosynthesis?. Phenology of Ecosystem Processes.

[39] Gaumont-Guay D, Black TA, Griffis TJ, Barr AG, Jassal RS (2006). Interpreting the dependence of soil respiration on soil temperature and water content in a boreal aspen stand.. Agricultural and Forest Meteorology.

[40] Rayment MB, Jarvis PG (2000). Temporal and spatial variation of soil CO_2_ efflux in a Canadian boreal forest.. Soil Biology and Biochemistry.

[41] Drewitt GB, Black TA, Nesic Z, Humphreys ER, Jork EM (2002). Measuring forest floor CO_2_ fluxes in a Douglas-fir forest.. Agricultural and Forest Meteorology.

[42] Khomik M, Arain MA, McCaughey JH (2006). Temporal and spatial variability of soil respiration in a boreal mixedwood forest.. Agricultural and Forest Meteorology.

[43] Drake JE, Stoy PC, Jackson RB, Delucia EH (2008). Fine-root respiration in a loblolly pine (*Pinus taeda* L.) forest exposed to elevated CO_2_ and N fertilization.. Plant, Cell and Environment.

[44] Sommerkorn M (2008). Micro-topographic patterns unravel controls of soil water and temperature on soil respiration in three Siberian tundra systems.. Soil Biology and Biochemistry.

[45] Ball BA, Virginia RA, Barrett JE, Parsons AN, Wall DH (2009). Interactions between physical and biotic factors influence CO_2_ flux in Antarctic dry valley soils.. Soil Biology and Biochemistry.

[46] Pregitzer KS, King JS, Burton AJ, Brown SE (2000). Responses of tree fine roots to temperature.. New Phytologist.

[47] Lin G, Rygiewicz PT, Ehleringer JR, Johnson MG, Tingey DT (2001). Time-dependent responses of soil CO_2_ efflux components to elevated atmospheric [CO_2_] and temperature in experimental forest mesocosms.. Plant and Soil.

[48] Cheng WX, Johnson DW (1998). Elevated CO_2_, rhizosphere processes, and soil organic matter decomposition.. Plant and Soil.

[49] Pregitzer KS, Zak DR, Curtis PS, Kubiske ME, Teeri JA (1995). Atmospheric CO_2_, soil nitrogen and fine root turnover.. New Phytologist.

[50] Pregitzer KS, Zak DR, Maziasz J, DeForest J, Curtis PS (2000). Interactive effects of atmospheric CO_2_ and Soil-N availability on fine roots of *Populus tremuloides*.. Ecological Applications.

[51] Matamala R, Schlesinger WH (2000). Effects of elevated atmospheric CO_2_ on fine root production and activity in an intact temperate forest ecosystem.. Global Change Biology.

[52] Lin GH, Ehleringer JR, Rygiewicz PT, Johnson MG, Tingey DT (1999). Elevated CO_2_ and temperature impacts on different components of soil CO_2_ efflux in Douglas-fir terracosms.. Global Change Biology.

[53] Andrews JA, Schlesinger WH (2001). Soil CO_2_ dynamics, acidification, and chemical weathering in a temperate forest with experimental CO_2_ enrichment.. Global Biogeochemical Cycles.

[54] Steinmann K, Siegwolf RTW, Saurer M, Körner C (2004). Carbon fluxes to the soil in a mature temperate forest assessed by ^13^C isotope tracing.. Oecologia.

[55] Buchmann N (2000). Biotic and abiotic factors regulating soil respiration rates in *Picea abies* stands.. Soil Biology and Biochemistry.

[56] Morén A-S, Lindroth A (2000). CO_2_ exchange at the floor of a mixed boreal pine and spruce forest.. Agricultural and Forest Meteorology.

[57] Wieser G (2004). Seasonal variation of soil respiration in a *Pinus cembra* forest at the upper timberline in the Central Austrian Alps.. Tree Physiology.

[58] Subke J-A, Reichstein M, Tenhunen JD (2003). Explaining temporal variation in soil CO_2_ efflux in a mature spruce forest in Southern Germany.. Soil Biology and Biochemistry.

[59] Davidson EA, Belk E, Boone RD (1998). Soil water content and temperature as independent or confounded factors controlling soil respiration in a temperate hardwood forest.. Global Change Biology.

[60] Yuste JC, Janssens IA, Carrara A, Ceulemans R (2004). Annual Q_10_ of soil respiration reflects plant phonological patterns as well as temperature sensitivity.. Global Change Biology.

[61] Xu M, Qi Y (2001). Soil-surface CO_2_ efflux and its spatial and temporal variations in a young ponderosa pine plantation in northern California.. Global Change Biology.

[62] Cardon ZG, Hungate BA, Cambardella CA, Chapin FS, Field CB (2001). Contrasting effects of elevated CO_2_ on old and new soil carbon pools.. Soil Biology and Biochemistry.

[63] Pendall E, Bridgham S, Hanson PJ, Hungate B, Kicklighter DW (2004). Below-ground process responses to elevated CO_2_ and temperature: a discussion of observations, measurement methods, and models.. New Phytologist.

[64] Schimel JP, Gulledge JM, Clein-Curley JS, Lindstrom JE, Braddock JF (1999). Moisture effects on microbial activity and community structure in decomposing birch litter in the Alaskan taiga.. Soil Biology and Biochemistry.

[65] Mielnick PC, Dugas WA (2000). Soil CO_2_ flux in a tallgrass prairie.. Soil Biology and Biochemistry.

[66] Saiz G, Byrne KA, Butterbach-Bahl K, Kiese R, Blujdea V (2006). Stand age-related effects on soil respiration in a first rotation Sitka spruce chronosequence in central Ireland.. Global Change Biology.

[67] Nsabimana D, Klemedtson L, Kaplin BA, Wallin G (2009). Soil CO_2_ flux in six monospecific forest plantations in Southern Rwanda.. Soil Biology and Biochemistry.

[68] Xu W, Wan S (2008). Water- and plant-mediated responses of soil respiration to topography, fire, and nitrogen fertilization in a semiarid grassland in northern China.. Soil Biology and Biochemistry.

[69] Guan DX, Wu JB, Zhao XS, Han SJ, Yu GR (2006). CO_2_ fluxes over an old, temperate mixed forest in northeastern China.. Agricultural and Forest Meteorology.

[70] Zheng JQ (2008). Effects of elevated concentrations of atmospheric CO_2_ on soil microbial community..

